# Intrinsic Motivation and Psychological Connectedness to Drug Abuse and Rehabilitation: The Perspective of Self-Determination

**DOI:** 10.3390/ijerph16111934

**Published:** 2019-05-31

**Authors:** Gloria H. Y. Chan, T. Wing Lo, Cherry H. L. Tam, Gabriel K. W. Lee

**Affiliations:** 1 School of Social Science, Caritas Institute of Higher Education, Hong Kong, China; hychan@cihe.edu.hk; 2 Department of Social and Behavioural Sciences, City University of Hong Kong, Hong Kong, China; ss.hltam@cityu.edu.hk (C.H.L.T.); gabriel.lkw@my.cityu.edu.hk (G.H.W.L.)

**Keywords:** drug addiction, drug relapse, psychological need, relatedness, self-determination Theory

## Abstract

This study adopts the perspective of the Self-Determination Theory to look at the psychological experience of drug users and their decisions to take drugs or not, with particular emphasis on the concept of relatedness. To achieve this objective, a qualitative methodology was employed to explore the experiences of these drug users regarding how they take drugs and/or relapse. Theory-driven thematic analysis was employed to identify themes related to this topic. Results show that one’s psychological need for relatedness is an important determinant of whether one will take drugs or not, via the interaction mechanisms that exist in dimensions of affiliation and intimacy. While drug taking is a result of the modeling behavior existing in affiliated relationships, it is also a coping strategy for the ultimate satisfaction of psychological needs when human relatedness disappears. The implication is that significant others can develop unconditionally warm, caring, and empathetic supportive relationships with drug users, so as to enhance their fulfillment of psychological needs and reduce the risk of drug relapse.

## 1. Introduction

Drug addiction refers to the abusive, maladaptive dependence on drugs, which causes significant impairment or distress [1]. Existing literature has largely illustrated the causes of drug abuse and drug relapse. Regarding drug abuse, it is mentioned that the reasons for it can be categorized into individual factors and social/environmental factors [2,3,4]. Individual factors include low self-control [5], curiosity [6], pursuit of excitement and comfort [6], alleviation of distress [6], poor coping mechanisms with stress [3,7], and a low level of awareness of the negative consequences of drug abuse [6]. Social or environmental factors include the existence of life stress events [7], weak social bonds [3,8], and adverse environmental factors (e.g., bad role models such as negative peer influence and poor family education, low availability of rewarding life choices other than drugs, family relationship with little emotional contact) [2,3,6,9,10,11]. With respect to drug relapse, its reasons include both individual (e.g., low level of self-efficacy, negative perception of events) and social/environmental factors (e.g., existence of additional crises, lack of community support) [9,12,13,14,15]. When compared with social/environmental factors, individual reasons for drug relapse should be regarded as equally important [6]. For instance, when former drug users have difficulty in engaging in normal family life and social interactions (e.g., experiencing disconnection from the family, divorce, and social exclusion) and they fail to cope with these stressful events adaptively, they are likely to relapse [16,17]. In all, what causes drug use and drug relapse involves a dynamic interplay of personal and contextual factors.

In Chinese communities, Chinese are known to value *guanxi* (social connectedness or relationships). *Guanxi* has been described as tight, close-knit networks, interpersonal connections, particularistic ties, friendship, and reciprocal exchange [18,19,20,21,22,23]. *Guanxi* is a special cultural characteristic that impacts interpersonal behavior in every Chinese community. Chinese people try to subtly nurture *guanxi*, as it affects their attitudes towards the development and maintenance of long term social relationships [24]. The development of *guanxi* starts with a *guanxi* base that is formed through ascribed base (e.g., kinship) or social base (e.g., school or workplace). The Chinese people “must interact, exchange favors, build trust and credibility, and work over time to establish and maintain the relationship” [24] (p. 208). Thus, they are psychologically motivated to achieve the *guanxi* embedded in their daily lives. However, relatively less literature has investigated why Chinese people living in metropolitan cities take drugs and/or experience drug relapse in the light of the fulfillment of psychological needs from the perspective of social connectedness and relatedness. Hence, this study seeks to fill the research gap by investigating this topic using Self-Determination Theory (SDT) [25], which illustrates how one’s intrinsic motivation and psychological needs are related to one’s drug-taking behavior. In the present study, SDT is used because it not only includes the extrinsic motivators (e.g., outside pressure and control, external rewards) on one’s behavior, but also the effects of intrinsic motivators (e.g., the role of connectedness, belonging, and social support in recovery).

### 1.1. Self-Determination Theory

SDT assumes that human beings have an inborn tendency to pursue growth, well-being, and health. To encourage a person to pursue a particular goal, there are broadly two kinds of motivation: intrinsic and extrinsic motivation. Intrinsic motivation refers to a person’s engagement in a particular behavior for their own sake (e.g., such behavior is personally rewarding and enjoyable for them), while extrinsic motivation refers to one’s engagement in certain behavior because of external outcomes (e.g., monetary rewards, awards, and social recognition) [26]. Specifically, there exists a motivation continuum in which the types of motivation range from amotivation to intrinsic motivation [27]. Amotivation means the lack of motivation to act because of: (1) the lack of efficacy and/or perceived competence to attain the desired outcomes; and/or (2) the inability to see the value of performing particular activities. Extrinsic motivation is further divided into four types of regulation: external regulation (i.e., behavior performed due to outside control and demand), introjected regulation (i.e., behavior performed based on the avoidance of feelings of shame, guilt, and/or obligation but not internal acceptance), identified regulation (i.e., behavior performed because one values such action as important), and integrated regulation (i.e., behavior performed due to the satisfaction of an external reason, while finding such behavior important and coherent to own values and goals). For intrinsic motivation, this refers to performing actions solely based on one’s own enjoyment, satisfaction, and self-interest instead of external rewards [27]. In order to enhance one’s persistence in certain actions, it is highly encouraged that the locus of causality and control should move from external to internal. In other words, motivations should be more self-determined, integrated, and intrinsic [28].

To enhance one’s autonomous integration and/or intrinsic value of certain behavior, internalization is important [26]. To facilitate the process of internalization, the fulfillment of psychological needs is essential [25,26]. In SDT, a psychological need is regarded as a basic, innate desire, “an energizing state” [27] (p. 74), which significantly predicts the optimal function, development, and well-being of individuals [25], “but if not satisfied, contributes to pathology and ill-being” [27] (p. 74). There are three types of essential psychological needs in SDT: autonomy, competence, and relatedness. Autonomy refers to one’s active choice for particular actions and the willingness to perform particular actions intuitively based on the consistency of the actions with one’s sense of self. Competence refers to the ability to exert changes, master the environment, and develop new skills to bring about desired outcomes. Relatedness refers to the need to be connected with others, accepted by others, to love and provide care for others as well as to be loved and cared for by others [25,29]. This need can be satisfied when one perceives oneself as being included in a group, experiences belongingness to a group or community, and has close relationships with others [25]. Although relatedness is sometimes considered as less important than the other two needs (e.g., a person can be intrinsically motivated to do something solely based on autonomy and competence), the absence of relatedness (e.g., loss of secure relational attachments to significant others like parents) likely leads to a failure in the emergence of intrinsic motivation [25]. Given that relatedness is essential, it is important to find out how one’s relationship to significant others (e.g., family, peers, and spouse) helps them to achieve positive, optimal development [30,31].

### 1.2. Application of SDT in the Context of Drug Use and Drug Relapse

According to SDT, autonomous self-regulation (i.e., motivation that is intrinsic, identified, or integrated) is important for promoting healthy behavior because it helps one engage in performing such behavior and in making the behavior sustainable [27]. To help enhance one’s motivation to make positive changes, both the personality (i.e., individual level) and the context (i.e., social/environmental level) are essential. At the individual level, for example, people can be encouraged to explore the environment autonomously so as to foster their autonomous self-regulation [26]. At the social/environmental level, autonomy support/need support, which “involves encouraging others to be self-initiating rather than pressuring them to behave in a particular way,” is important (e.g., the practitioner taking the perspective of the service users and understanding their feelings, giving them information support and emotional support) [32] (p. 1114). The sole existence of the individual level is insufficient. Autonomy support/need support must exist in the social/environmental context (i.e., one must perceive that the three basic, innate psychological needs are satisfied) to encourage healthy development and well-being, or else one’s development of autonomous self-regulation and the internalization process will be blocked [25,26,32].

Applying SDT to the context of drug abuse and drug relapse, it is postulated that solely providing external motivators (e.g., parental pressure) is not sufficient for one to quit drugs, because one may engage in treatment out of fear of any possible consequences, but not because of an internal sense of control and autonomy [33]. Instead, building good relationships with the drug users and offering them autonomy support/need support (e.g., respecting their choices with respect to treatment; providing a safe environment for them to practice adaptive behavior; offering them acceptance, warmth, understanding, support, and unconditional positive regard) may help facilitate their internalization and autonomous self-regulation. This helps foster their attainment of the goal to quit taking drugs based on intrinsic motivation [25,34]. According to [35], service users of a methadone maintenance program were more engaged in the program and experienced lower occurrence of drug relapse when they were highly intrinsically and autonomously motivated. While both high internal and external motivations predicted optimal treatment outcomes, the sole existence of external motivation negatively predicted adherence to the program, reflecting that coercion or external forceful pressure is not useful in helping someone to stop taking drugs.

Summarizing the above literature, it is known that intrinsic motivation is a fundamental incentive driving individuals to direct behaviors. The same applies to drug users. To nurture their intrinsic motivation, the provision of social context is central. Such context should provide support for their autonomy, competence, and relatedness so as to encourage them to move towards healthy development and well-being [25,26]. For example, when they perceive that: (1) their viewpoints, choices, feelings, and emotions are understood, acknowledged, and supported without judgment (e.g., their difficulties in dealing with stress and the drug cessation process are understood by another person); (2) the advice given by others is backed up by an underlying rationale rather than exerted in the form of control and pressure; (3) there are a number of effective strategies available for making changes; (4) their positive changes (e.g., giving up drugs) are relevant to their personal aspirations in life; (5) they can succeed in making positive changes (e.g., giving them unconditional regard; helping them understand that failures pave the way to future successes); and (6) the plans for quitting drugs are appropriately designed to fit their needs and level [26]. In other words, when they perceive themselves as efficacious in quitting drugs, and more importantly, receive empathetic understanding, as well as unconditional warmth, love, support, care, and accompaniment in relationships (i.e., the satisfaction of psychological needs), it is likely that they will enjoy a higher sense of well-being and become intrinsically motivated to move towards healthy behavioral patterns (e.g., quitting drugs) [26]. To the contrary, without significant others (e.g., due to separation or death), their drive towards healthy development will be thwarted because they experience loneliness and/or other negative emotions.

### 1.3. Insights from the Literature

**Figure 1:**
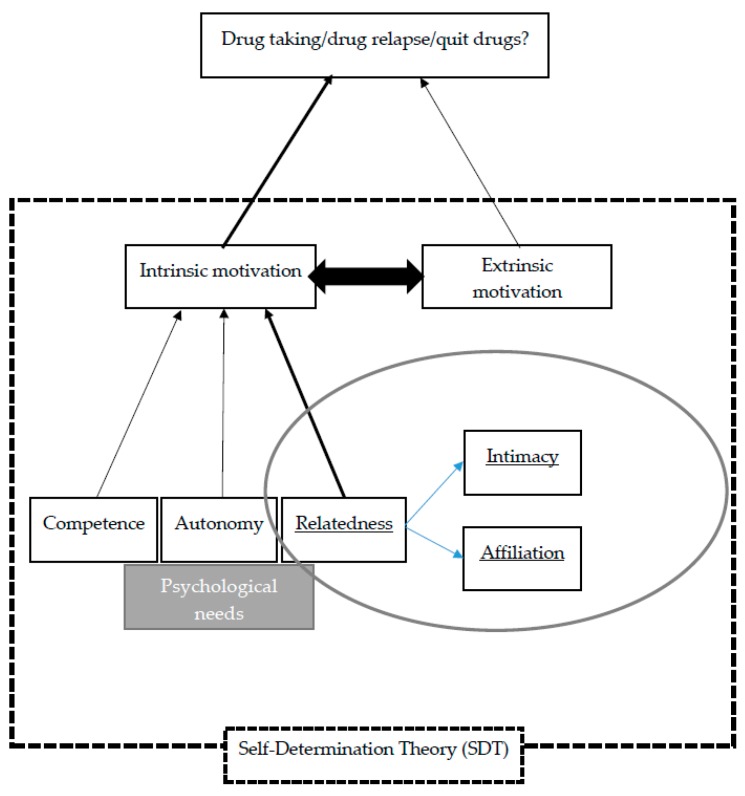
Theoretical framework for thematic analyses.

Much of the literature about the reasons for drug taking or drug relapse has focused on factors such as self-control [5], poor coping mechanisms [3,7], negative peer pressure [4], psychological responses [11], and lack of awareness of the negative consequences of taking drugs [6,36]. From the perspective of SDT, the drug users’ psychological experience is a crucial factor determining whether they will turn to drugs or quit them. The fulfillment of the psychological needs constitutes the internal motivation of drug users which drives them to quit drugs and pursue healthy development. As shown in Figure 1, the three types of psychological needs are inter-related because drug users’ autonomy and competence in quitting drugs can be fostered through warm, supportive relationships with empathetic understanding and unconditional positive regard (i.e., relatedness). For instance, clients will feel they have the ability to quit drugs successfully and that they have choices in participating in a treatment program in a non-coercive environment [25,26,27]. Relatedness can be understood in terms of two aspects: affiliation (i.e., “the tendency to avoid loneliness by seeking the company of others”) and intimacy (i.e., “the tendency to experience warm mutual exchange in interpersonal relationships”) [37] (p. 1148). Based on these concepts, this study seeks to explore the following areas: (1) how people’s drug-taking behavior is related to their wish to build and maintain relationships with others; and (2) how people’s drug-taking behavior is related to their loss of significant others who brought them a sense of connectedness, warmth, comfort, and security, as well as spiritual support to cope with challenges in daily life. The loss of human relatedness brings them loneliness and emptiness, which drives them to take drugs as compensation. Enquiring into the drug users’ narratives might also facilitate their recovery—the process in which the drug users review and reinterpret their drug-taking and drug-quitting experiences can serve as an integral process of recovery as it helps them reconstruct their sense of self and establish a drug-free identity [38].

### 1.4. Present Study

This study investigates the importance of intrinsic motivation in drug rehabilitation, in the satisfaction of the drug user’s psychological needs, including their willingness to perform particular actions (autonomy), the sense of having the capacity to develop new skills and master the environment (competence), and sense of connectedness to love and to be loved (relatedness). To achieve the research objective, this study adopted a qualitative research methodology to explore the experiences of people who engage in drug abuse and experience drug relapse, so as to understand their psychological experience in choosing to take drugs and/or to quit drugs.

## 2. Methodology

### 2.1. Procedure and Participants

This qualitative study involved a sample of 103 drug users. The participants were drug users who were detained and received treatment in four government-run treatment centers in Hong Kong, or under community supervision, under the Drug Addiction Treatment Centers Ordinance (Cap, 244). After the ethical review was approved by the Research Ethics Committee of City University of Hong Kong (9211123), data collection was conducted in the second half of 2017, facilitated by the Correctional Services Department of Hong Kong. The purposive sampling method was used to ensure a fair distribution of inmates, supervisees (those under post-released community supervision) and recallees (those who are recalled to in-center treatment after they relapse during post-release community supervision) selected from the four institutions.

Data were collected through individual interviews and focus groups conducted by a team composed of a researcher and a research assistant. There were 67 participants who took part in a one-to-one, semi-structured interview (see Appendix A) for around 30 min to one hour. In addition, six 1.5-h long focus groups, each with six inmates, were run with a total of 36 participants. The process and experience of drug abuse and drug relapse were explored. Both interviews and focus groups were audio-recorded to facilitate transcription into Chinese. During transcription, names of interviewees were changed to codes. For the purpose of this paper, selected verbatim were translated into English, and the translation was confirmed by two investigators. Occasional grammatical corrections were conducted without sacrificing content precision.

### 2.2. Analyses

Theory-driven thematic analysis was adopted to analyze the data based on the literature review, which informs the research objective. The analyses of the data were performed based on the following procedure: (1) “familiarizing with the data”; (2) “generating initial codes”; (3) “searching for themes”; (4) “reviewing themes”; (5) “defining and naming themes”; and (6) “producing the report” [39] (pp. 87–93). To begin with, data were studied by two researchers in order to enhance the familiarity with it. Then, codes were assigned by the two researchers to enhance the inter-rater reliability, and made to link up theoretical ideas with the data (see Figure 1 for the theoretical framework). After the review of the data and refinement of the codes to ensure their consistency, themes were generated based on the theoretical concepts illustrated in the literature, such as psychological needs and relatedness. Based on the nature of the categorized data under each theme, subthemes were developed and modified, in order to capture the linkage of the data to the literature and theories. During analysis, a word search function was performed in Microsoft Word, which helped find the codes and themes based on the frequency of keywords or repeated ideas expressed by the participants [39].

## 3. Results

### 3.1. Background of Participants

Among the 103 participants, 50% were male and 50% female. They fell into the age range of 11−70 years old, in which the age group of 21–30 was the largest (*N* = 41, 40.2%) and the age group of 51 and above was the smallest (*N* = 6, 5.9%). More than half of them (*N* = 66, 65.3%) took drugs more than six times a week. Regarding the types of drugs taken, the participants could report more than one option; among the different types of drugs, crystal methamphetamine (nicknamed ‘ice’ or ‘meth’) was the most popular (67%). Regarding their experience of drug taking, most started taking drugs at the age of 11−20 (*N* = 77, 77.8%). In terms of the duration of their drug taking, most of them had taken drugs for more than 10 years (*N* = 49, 49%), and the most important reason for taking drugs was given as peer influence (see Table 1 and Table 2).

### 3.2. Introduction of the Themes

Based on the framework of SDT, the theoretical concepts (e.g., intrinsic motivation, competence, autonomy, relatedness, extrinsic motivation) were selected as themes to guide the search of meaningful data (Table 3). Data about the reasons for taking drugs and quitting drugs was extracted according to the following coding structure, so as to uncover the drug recovery experiences of the participants.

### 3.3. Analyses of the Themes and Quotes

#### 3.3.1. Relatedness with Significant Others

Among the participants, many took drugs initially because of the influence of negative emotions (*N* = 21). They felt unhappy and sad (*N* = 10). For example, as NS4 said, “At that moment I was upset and discouraged…I don’t know how to describe how discouraged I was…”. One of the key sources of their negative emotions came from the poor relationships with their significant others. Therefore, a key function of drug taking was psychological substitutes for the lack of connectedness in their everyday life.

#### 3.3.2. Psychological Substitutes for the Lack of Connectedness in Life

There were 17 participants who mentioned that having poor relationships with significant others enticed them to take drugs. Some had poor relationships with their family (*N* = 14) (e.g., NR2: “When I was small, I felt that my family treated me poorly”). Apart from not having a harmonious family (e.g., NS6: “I had a poor relationship with my father before”; LKR1: “My family is complete, but my family members have a generation gap. My father is much older than my mother…also I had a strict upbringing…and my father is quite stubborn…he also had a daughter and a son with another family…so he didn’t care much about me”), they experienced parental divorce (e.g., NR4, HF2, NS5) as well as subsequent conflicts with stepfathers (e.g., LKS1) and stepmothers (e.g., NS10). Not being able to receive warmth and comfort from these significant others, they took drugs as a coping strategy. In contemporary society, parents usually work and their lives are hectic, leaving little quality time and sufficient communication with their children. According to the participants (*N* = 7), the lack of care and support received from the significant others was also a key reason to resort to drugs. As their parents seldom communicated with them, they could not feel love and care in the family (e.g., NS4, LSF1, NR7) (e.g., LSF1: “My parents always ignored me because of work…”; NR7: “The type of ‘love’ that they gave me was…they wouldn’t spend time with me, but kept on giving me money…”). When they suffered from negative emotions and failed to have support from significant others (e.g., NR7: “…I felt very upset at that time, because I couldn’t find anyone to talk to in my family. Only drugs can hypnotize me…”), they would turn to drugs for comfort instead.

Apart from parents, some participants suffered from a poor relationship with their spouse (*N* = 6). NR1 recalled that “at the time when I was pregnant, my boyfriend asked me to marry him, but he told me to abort the child the next day. I collapsed when I heard his request.” Similar kinds of sudden blows were hard and heartbreaking for the participants. Before the breaking up of a relationship, participants might have been tolerating their situation for a certain period of time (e.g., NS1: “My husband had an affair. I had been tolerating this for a long time”; NS7: “My ex-husband left me. He thought that I was too fat and didn’t know how to do housework. I tried to lose weight but I couldn’t manage to do so”). From the above verbatim accounts, it is seen that participants experienced rejection from their spouse based on conditions (e.g., physical appearance) and had to fulfill the requirements of their spouse at the expense of own wishes (e.g., aborting the baby). Not having significant others who could offer them unconditional love, care, and acceptance, they took drugs to alleviate the negative emotions (NR1: “I felt heartbroken, so I took drugs”).

Loss of spouse/companions also led to them relapsing easily (*N* = 11). Getting divorced (*N* = 7) or breaking up with a spouse (*N* = 4) made participants feel “down” (HR1), “emotions went ‘rock-bottom’”, and “in complete despair” (HF1). For instance, HM6 shared that “When I completed my drug treatment, I was happy…but I discovered that my wife had an extra marital affair…so I took drugs again…to hypnotize myself”; NM3 also shared that “At that time I had a boyfriend. I had waited for him for three years. After he had been released from the prison, he got back with his ex-girlfriend. So my time was wasted on him. I collapsed right away.” This shows that the relationship problems with a spouse/companions were traumatic for them. Hoping to alleviate their negative emotions, they took drugs to “hypnotize themselves” (HM6).

#### 3.3.3. As a Way to Maintain a Relationship or Affiliation

On the other hand, some participants took drugs because they were in normal or even good relationships with significant others. Five participants expressed that their families were also drug takers, so they had easy access to drugs. Some even mentioned that the first drugs they took were provided by their family (e.g., HR5: “The drugs that I first took were given to me by my brother”). Among participants whose families had a history of drug taking, family members who had drug-taking experience were usually their parents, siblings and relatives (e.g., HF2). Not having the choice to break the relationship with their family, they had more chances of becoming involved with drugs. Seven participants took drugs under the influence of their spouse. They described how “he took drugs, so I took drugs too” (NS10); “I took drugs all because of my husband” (NS1). This shows that they could hardly escape when their spouse also took drugs. NR1 mentioned that “My boyfriend took drugs too. He is the father of my daughter. I don’t want my daughter to lose her father, because the previous one has already lost her father. It’s not desirable not having parents when you are young and growing up in a single-parent family.” For the sake of maintaining the relationship and keeping the family together, she chose to take drugs too. As a result, drug taking was desperately employed as a way to maintain a relationship.

A number of participants took drugs due to peer influence (*N* = 65). Some had poor family relationships and thus got involved with drugs under peer influence. For example, as expressed by HF2, “because I was born in a single-parent family, and maybe you’ve only the father or the mother, s/he will then have no time to spend with you. So, I went out on the streets and made friends. We then started to play together”; similarly, NR5 said that “at that time, I played with the friends next door, and I was not in a good relationship with my family, so I went out and found my friends. They suggested playing together and taking drugs together. Then I became addicted to drugs.” This suggests that when youngsters do not feel care from the family, as an alternative they will turn to friends for care and comfort. Being friends with drug-taking peers induced participants to take drugs and develop the habit of drug taking; such peer influence was stronger if the friends were important to the participants. For example, NR1 expressed that “I did think of quitting drugs, but everyone around me was taking drugs, plus I didn’t have enough determination to do so. So, I relapse very easily and it’s hard for me to break out of the circle.”

Likewise, NM3 explained that “as I grew up in that place, and all the people around me are the same type of people, it’s easier for me to access drugs…they’re my friends whom I’ve known since I was young. Who else I can turn to?” As they still had contact with drug-taking friends, even if they wished to quit drugs, it was not easy for them to get out of such peer groups and persist with their wish to quit drugs. Besides, drugs were important media for participants to maintain friendships. For example, some participants expressed that “if all of them took drugs but I walked away, it would look like I was isolated from them and I would not be able to get into their circle” (HR4); likewise, as expressed by LSS3, “sometimes it was unreasonable if my friends wanted to take drugs but I didn’t go with them. So I followed them and took drugs with them.” This shows that they take drugs because they wanted to avoid being alienated and they wanted to be one of the members of the peer group.

In summary, the verbatim accounts show that participants appear to use drugs as an alternative solution to alleviate negative emotions and regain a positive sense of connectedness. Such a sense of connectedness can originally be achieved when receiving unconditional care and support from significant others (e.g., family and spouse), as well as peers sharing similar life goals; however, they fail to receive these in life, resulting in feelings of distress and loneliness. When being able to handle these negative emotions, the likelihood of them resorting to drugs in search of comfort and a sense of satisfaction increases. On the other hand, a high susceptibility to the influence of family, spouse, and friends reflects their need for interpersonal connections; they develop the habit of drug taking either due to modeling (e.g., following what the drug-taking family members and friends do) or a wish to stay connected with other people (e.g., not wanting to lose significant others).

#### 3.3.4. Significant Others are Usually a Double-Edged Sword

Apart from the negative influences, receiving care and support from significant others was one of the motivators for participants to quit drugs (*N* = 9). This could be achieved from the family (*N* = 8) and the spouse (*N* = 1). Some participants expressed that they quit drugs because of the love from their family (e.g., LKF1: “My family loves me very much”; NS7: “My mother told me to quit drugs and said the whole family would accept me again and let me go back home. This was my motivation to quit”). They did not wish their families to be worried about them and get hurt (e.g., LSF1: “I don’t want my family getting hurt and sad…they were in tears when visiting me [in the treatment center]”), so they decided to quit drugs. This shows that the existence of significant others can be a double-edged sword [40]. The feelings of distress and loneliness from the lack of connectedness with family can easily drive the participants to resort to drugs, while unconditional love, support, and acceptance from significant others are crucial for the participants to gain comfort and strength to resist against drugs.

Some participants even regained the motivation to achieve drug abstinence forever when they perceived the care and support from significant others as the powerful force driving them to quit drugs (*N* = 48). For example, NR2 shared the experience of receiving unconditional love and support from the family: “My mother came to visit me in the Drug Addiction Treatment Center regularly on holidays and she didn’t give up on me. She didn’t ignore me due to my drug-taking behavior; rather, she hid the secret from relatives and protected me from being looked down on by them. So my motivation [to quit drugs] comes from my mother.” This shows that the continuous support from the family helps strengthen the will to quit drugs. LSS2 also expressed that “when you are admitted into the Drug Addiction Treatment Center, it’s just like you’re no longer backed up by friends…no people contact you…but my family came to visit me once a week, every week, four times a month…I think no one else would treat me like they’ve done.” Participants reflect that the friends who they used to be greatly concerned about were not as reliable and trustworthy as their own families; their families become a source of motivation, encouraging them to quit drugs and lead new lives. As illustrated by NS7, “When you have your family, you’ll have hope; if you have no family, you’ll lose the motivation to live…I need to treat my parents better but not let them down again.” For the participants, family members who love and support them unconditionally are important significant others who can highly motivate them to give up taking drugs.

Another motivator for achieving drug abstinence permanently is the loss of significant others (*N* = 3). The passing of their family members (*N* = 2) helped them experience an epiphany. As NS2 said, “When I went to the drug treatment center this time, my family member passed away…I cannot see him again when I’m discharged…I know that my grandfather died…he loved me most…I cannot risk having/doing something else that I would regret.” Losing significant others brought about regret; wishing not to have a similar experience again, participants were motivated to quit drugs on a permanent basis. Also, losing significant others helped participants understand that they could count on no one but themselves in order to beat their drug abuse; as shared by NM1, “Mother…I loved her very much; at that time, she gave me money for buying drugs every day…now I wish to quit drugs very much because I knew that she wanted me to do so. Now she’s gone, I know that I have to rely on myself, not my family.” In spite of experiencing a cut-off of their relationships with significant others, the spiritual connections still existed, in which the participants could find strong spiritual support to lean on and keep on with their motivation to quit drugs.

#### 3.3.5. Connectedness with God

Another connection that is significant in driving the participants to quit drugs is having a religion (*N* = 7). Through the power of religion and the link to the church, participants started to adopt positive thinking. For example, NS7 felt that “I can atone for my crime and turn bad things into good things.” This shows that religion also serves as a spiritual support to which participants can attach themselves so as to find the psychological support to resist drugs. They believed that religion was a kind of “spiritual sustenance” (HS4), which could help them stay away from drugs. For example, NM5 believed that “when you have religious beliefs, you’ll be determined to quit drugs, like you’ve got a goal to achieve.” This reflects that for some participants, they can rely on and establish a spiritual connectedness with religion, so that they can find the comfort and sense of contentment to sustain their will to quit drugs [25].

#### 3.3.6. Sense of Competence and Connectedness to Work

A lack of life goals and sense of self-worth is another dimension that leads to drug abuse and relapse. Some participants found that they did not have own life goals (*N* = 10), feeling that there is “nothing to do” (e.g., LSR1), “bored” (e.g., HF1, NS9), and “dull” (e.g., HR1) (e.g., NR1: “I’m afraid of being bored…I don’t know how to spend my time…this happens to many housewives…then I want to take drugs as a way to make time pass faster”; HM8: “I seldom go out, after all; I return home immediately after work. When I don’t take drugs, I really don’t know what I can do”). With a lack of life goals and competence in work, their lives became more routinized and they did not have something that they could depend on in order to feel satisfied; as a result, they took drugs as pastime. When they got used to taking drugs as a way to alleviate boredom, gradually, it became an essential, indispensable part of their lives, which became increasingly difficult to get rid of. As NM2 said, “I don’t know what to say. It’s just like you need to eat every day. When the time comes, you’ll do such a thing (drug taking).”

When life goals were clear, this helped participants to quit drugs (*N* = 3). For example, NR6 expressed that “Then we both went to work, and worked hard. Later we had a child (elder daughter)…actually we planned to get married at that time…yes, we had bought a flat together and built our home (The participant quit drugs when she was pregnant at that time),” while HF2 mentioned that “I need to earn money and I have no time (to take drugs),” because his spouse was pregnant. These life events and goals not only encouraged participants to resume a “normal” life, but also helped them regain hope for the future and reduce their intention to take drugs to seek short-term pleasure or satisfaction. These constitute the sources for psychological sustenance that the participants can depend on in order to remove the need for drugs to seek comfort and satisfaction.

Having found a job and having a sense of competence and satisfaction also encouraged participants to quit drugs (*N* = 10). They believed that work could make life more fruitful and meaningful. For example, as expressed by HF2: “I don’t think about such things (drugs) when working. I’m very self-disciplined. Like when I’m working in the warehouse, I don’t have time to think about drugs. I’ll only think about it after work”; likewise, NM1 expressed that “after leaving the drug treatment center, if I didn’t go to work and had nothing to do, I would easily turn to drugs again. So, this (going to work) is very important.” This shows that, on the one hand, work occupies the participants’ daily lives and distracts them, thus they experience a lower drive to turn to drugs to seek spiritual support. On the other hand, work also helped the participants to achieve a sense of competence and fulfillment. For example, NM2 expressed that “at that time, when I could totally keep away from drugs, I just got a job and the job was fulfilling.” NS10 also expressed that “I like to work. It gives me a sense of satisfaction.” This reflects that work not only serves as a new life transitional event, which structures their lives and prevents them from taking drugs again [41,42], but also it enhances their motivation to quit drugs when they have a stronger sense of mastery of their everyday life.

Of course, some participants (*N* =14) took drugs as a result of pressure from a high level of discipline from the family (e.g., HR7), work (e.g., NR6, HS12), or multiple sources (e.g., NM1: “Maybe because of the environment, friends, and work…”), which made them resort to drugs to seek comfort to alleviate their pressure and feelings of distress. They felt that drugs helped them calm down, described as “feeling the quietness” (e.g., NS4), and helped them temporarily forget the stresses of life: For instance, NM8 said that “I could forget everything when I took drugs,” whilst NR4 mentioned that “I knew that drugs could help me forget my troubles.” As a result, they used drugs as a way to escape from reality (e.g., HS5: “The feeling of having taken drugs was…I needed not worry anymore…maybe I wished to escape from the reality”).

#### 3.3.7. Autonomy—Willingness to Quit

Although severe discipline and strong social control (i.e., external regulation in extrinsic motivation) are usually treated as important in restraining people from delinquency, the participants felt pressure (e.g., HR7) and were hostile towards severe discipline (*N* = 4). They expressed that “It was just like hanging myself and I couldn’t breathe” (LSF1) and they even developed rebellious thoughts against the family (e.g., NR2: “The harsher and tougher the family treated me, the more rebellious I was. I didn’t want to go home”). Additionally, there were participants who were highly disciplined and restrained by their spouse, thus they lost their freedom and the social life that they used to have (e.g., NS1: “I used to have a lot of friends. After I knew him (boyfriend), there were so many restrictions, I couldn’t do this and that. Friends then isolated me. I couldn’t drink…even a little…neither could I wear make-up…”). Such pressure only drove participants to take drugs as a way of coping, implying that external regulation is not effective for achieving drug abstinence.

In contrast, many participants quit drugs based on their autonomous decisions (*N* = 12). For instance, some wished to quit drugs because they experienced their negative effects. They felt that drug taking ruined their relationship with family and friends (e.g., NS6, NR5), as well as brought about a negative influence on their physical and psychological well-being. NS8 said, “At that time I was sluggish and looked ugly. I didn’t dare to eat. I looked so ugly. As you know, (taking drugs) causes people to look pale and skinny, and suffer from bad moods.” Drugs imposed harm to physical and psychological health, making the participants feel “tired of it” (HS5) and wanting to get rid of the influence of drugs. All these reflect that participants quit drugs based on their intrinsic motivation; understanding the value of quitting drugs highly encourages them to do so [28].

Some participants quit drugs willingly because of their children (*N* = 8). For instance, NS4 mentioned that “since I had my baby, I haven’t taken drugs from then on.”; NM5: “Since I was pregnant, I haven’t wanted to take drugs again. I’m afraid that it will affect the children, so I was strongly determined to quit drugs.” There was even one participant who, because of this reason, had given up drugs as long as six years ago (NS2). This shows that motherhood enhances participants’ willingness to quit drugs because they want their children to be able to grow up in a healthy family. Many participants believed that if they have a strong will to quit drugs, they would be able to do it successfully (*N* = 46). Having the strong will was important for the participants; as observed by NF2, “actually quitting drugs depends on yourself…because no matter how long you’ve locked someone up for, if s/he still wants to take drugs, s/he will do that eventually. The own self is the biggest enemy.” This was supported by LKS2, “if you really have the will to count on yourself to stay away from drugs, actually you can do so. But if you say yes but mean no, this is meaningless.” This shows that one’s self-efficacy, persistence, and perseverance (i.e., competence) [25] are the key for them to achieve drug abstinence.

To summarize, participants are motivated to quit drugs due to: (1) relationships with significant others in the existing social circle, which offer strong care and support for them as well as serve as adaptive, protective social bonds that encourage drug desistence; (2) willingly developing new social circles and new ways of life, which replace the old ones; and (3) their strong autonomous will to quit drugs because they recognize the advantages of staying away from drugs. This reflects that participants wish to quit drugs out of their intrinsic motivation; such motivation originates from the relatedness with their significant others, as well as the autonomy and competence to develop a brand-new drug-free life in which they can find spiritual sustenance to displace the need to seek comfort from drugs [25,26]. Their intrinsic motivation to quit drugs is largely sustained by the personal attachment experience that they receive, such as the unconditional love, support, and acceptance of their significant others, and even things in life to which they attach meaning and significance to gain psychological sustenance.

## 4. Discussion

From the results, it is reflected that extrinsic motivation (e.g., parental coercive discipline and control) is not useful for participants and even drives them to engage in deviant acts like taking drugs [28]. The things that motivate participants to stay away from drugs the most include the significant others (i.e., *guanxi*) as well as a new lifestyle with work, clear life goals, and religion. All these people and parts of life not only structure their time use and serve as adaptive social bonds, which reduce their engagement in delinquent behavior [8,41,42,43], but also provide a sense of well-being (e.g., enhanced self-efficacy and sense of security), which displaces the craving for drugs. For some participants, even significant others who have died can provide the spiritual support that they can lean on to sustain their motivation to quit drugs. The warmth, care, love, and comfort that the participants receive from the significant others as well as life goals help them find comfort, satisfaction, and security to fill the psychological void and gain strength during adversity. This helps nurture their psychological needs, which enhance their intrinsic motivation to direct towards positive, adaptive behavioral patterns [25]. Therefore, once they lose these significant others and lack direction in life, and they lack the self-efficacy to deal with these losses, the experience of the loss of significant others will result in negative emotions (e.g., emptiness and loneliness), driving them to resort to drugs as an alternative way of relief. On the other hand, it is noted that relationships with significant others can both increase and decrease the likelihood to take drugs. Such relationships can reduce drug taking by serving as sources of spiritual support and adaptive social bonds, but can increase drug taking when the relational partners take drugs as well. Based on the importance of significant others in affecting participants’ decision on whether to take drugs or not, the above results will be discussed in two dimensions—namely intimacy and affiliation—of relatedness [29,37].

The need for psychological connectedness with other people has long been recognized as an innate need of human beings [25,44,45,46,47]. Such connectedness can be with people, organizations, and even religion, and it affects one’s emotions and behavior [29]. To fulfill this need, human beings tend to establish connections with other people and/or groups, until they have established a certain amount of relationships with other people to satisfy their needs for belongingness [29]. To help one achieve emotional well-being, these relationships, at best, should have the following characteristics: (1) frequent contact; (2) lower susceptibility to change; and (3) be mutually affective with care [29]. If not all of them are equally affective and close, at least these relationships should mainly help one avoid experiencing negative emotions; even if the relationships are not reciprocal, the positive effect of the relationships still works if one perceives that the other party has a care/affinity/liking for him/her [29]. In other words, people are in need of relationships with people with whom they wish to affiliate in the pursuit of belongingness and avoidance of negative emotions (e.g., loneliness) [37], as well as people to whom they attach meaning and importance for the sake of spiritual support and satisfaction [37].

Whilst relationships of affiliation help people achieve a sense of a shared group identity, which serve to elevate the chances of survival through building positive relationships that bring enjoyment, harmony, mutual exchange, and cooperation with others [48], relationships of intimacy are for achieving a higher sense of well-being through the profound, in-depth experience of warmth, mutual exchange, closeness, and connectedness [48]. Being successful in building and maintaining these relationships nurtures the psychological needs of people, helping them to experience positive emotions (e.g., sense of comfort and security) and to pursue a healthy development. In contrast, failing to achieve this need (e.g., experiencing rejection or cut-off of relationships, failure to establish meaningful relatedness with other people) will not only lead to distress (e.g., feelings of despair) and loneliness [49], but also lead to maladjustment and pathology in terms of psychological state and behavior, which drive one to crave for the need until it is satisfied again [29]. The loss of relationship partners does not necessarily lead to maladjustment and pathology; if the lost relationships are effectively substituted, the negative effects can still be overcome, and vice versa [29].

Applying this mechanism to the context of drug abuse and drug relapse, in terms of affiliation, a modeling effect is normally generated between partners, which makes them mimic each other’s behavior [50]. Hence, when significant others (e.g., friends) take drugs, those around them may take drugs too. When significant others (e.g., spouse) quit drugs, so too will those close to them. With respect to intimacy, if the absence of significant others in life (e.g., divorce, break-ups, lack of care and support received from the family) cannot be effectively replaced by other forms of social support (e.g., friends), one’s craving for drugs will increase as a form of compensation. Conversely, if they are able to receive spiritual support from significant others, work, life goals, religion, or anything that they view as important and spiritually fulfilling, their psychological needs will be nurtured, which increases their intrinsic motivation to quit drugs.

It was noted that even significant others who have died can also bring people the same level of spiritual support and fulfill their psychological need for relatedness. Such a mechanism can be further explained with the use of Attachment Theory [44,45,46,51]. Attachment Theory originally explained the emotional bonding between the parent and the infant [44,45,46]. From an evolutionary perspective, human beings have an inborn desire to develop connectedness with an attachment figure (e.g., caregiver) in order to receive the feeling of being cared for and loved, as well as to achieve a sense of safety and security, especially when facing threats [45,46]. When an infant is separated from the attachment figure, it will experience intense distress [45,46]. Extended to the context of adulthood to explain interpersonal relationships [52], attachment can be understood as the emotional bonding that connects persons [44,46]. Attachment figures can be any special persons who serve the functions of: (1) proximity; (2) providing sense of comfort, support, and protection; and (3) providing security for a person to explore when s/he is in need [51].

A person can have more than one attachment figure; as one grows older, the attachment figures may change too [51]. For example, during infancy, parents (i.e., primary caregivers) are likely regarded as the ones mainly performing the functions of attachment; but when one grows to adolescence or even adulthood, other persons may appear and take up this role, such as siblings, relatives, friends, teachers, colleagues, romantic partners, spouses, and even symbolic figures like God [51]. These attachment figures will be organized into a hierarchy of attachment figures [46,53], in which a particular figure is treated as the principal figure to whom one will mainly turn when one is in need of security and protection, whilst the others serve as subsidiary attachment figures whom one will only turn to when the principal figure is absent [51]. In case of the absence of the principal attachment figure (e.g., loss or death), one will either reorganize the hierarchy (i.e., “editing”) by establishing new attachments or upgrading the existing figures, or transfer the attachment with the lost principal figure into a symbolic one as an adaptation to separation distress [51] (p. 76). Nevertheless, the success of such transference is dependent on: (1) the significance of the principal attachment figure to the person; and (2) whether the new figures can provide the same attachment functions as the lost principal figure [51].

An implication to the context of drug abuse and drug relapse is when drug users fail to find suitable persons who can perform the same attachment functions when their significant others have left, the anxiety and insecurity experienced will likely drive them to become attached to drugs as an alternative solution to regaining a sense of comfort and security. However, if they are able to do the editing, or transference of the deceased principal figure into a spiritual figure successfully, they will still receive the spiritual support and comfort that is strong enough for them to resist against drugs.

## 5. Conclusions

Relatedness is an essential element that determines drug users’ choice to take or to quit drugs. It provides important social capital which facilitates the recovery from drug abuse. Once this need is satisfied and internalized as intrinsic motivation, one will enjoy a high sense of well-being and be encouraged to engage in healthy behavioral patterns, as well as pursue positive growth and development, instead of resorting to drugs [25,26,54]. Given the significance of relatedness, it is advised that drug rehabilitation programs should focus more on nurturing drug users’ psychological needs, with particular emphasis on enhancing the emotional connections between the drug users and their significant others. For instance, more peer-led groups can be organized, as the users can likely find support, acceptance, and encouragement from the peers who are former users and use their lived experience to demonstrate how to cope with the challenges during recovery [55]. Most importantly, the significant others can try to be more supportive, understand better the needs and perspectives of the drug users and give them more encouragement to help them withstand the difficulties experienced during drug withdrawal, rather than coercing them to quit drugs or even avoiding them. This will help the drug users enhance their volition, self-efficacy, and confidence, as well as restore their sense of well-being, which constitutes their intrinsic motivation to quit drugs. Once their psychological needs have been satisfied fundamentally, their craving for drugs will be largely reduced, which in turn reduces the occurrence of drug relapse.

One important limitation of this study arises from the choice of theoretical perspective for investigating the topic. Since this study adopts SDT as the theoretical framework for analyzing drug use and drug relapse, it cannot cover other social and environmental factors that affect people’s decision to take drugs and/or quit drugs. In future studies, these factors can be incorporated into analysis in order to generate a more comprehensive picture of the drug problem.

## Figures and Tables

**Table 1:** Demographic and drug use data of participants.

Variable	*n*	%
Gender (*n* = 103)		
Male	52	50.5
Female	51	49.5
Age (*n* = 102)		
11−20	18	17.6
21−30	41	40.2
31−40	24	23.5
41−50	13	12.7
51 and above	6	5.9
Frequency of taking drugs (*n* = 101)		
Less than once per month	1	1.0
Once per month	1	1.0
2−3 times per month	9	8.9
1−2 times per week	14	13.9
3−6 times per week	10	9.9
More than 6 times a week	66	65.3
Types of drugs taken (multiple options)		
Crystal methamphetamine (Ice)	69	67.0
Cocaine	36	35.0
Ketamine	33	32.4
Heroin	23	22.3
Nimetazepam	14	13.6
Cannabis	11	10.7
Ecstasy	10	9.7
Others (Triazolam, Methaqualone, cough medicine)	12	11.6
Age of first drug−taking (*n* = 99)		
11−20	77	77.8
21−30	15	15.2
31−50	7	7.1
Duration of taking drugs (*n* = 102)		
Less than 3 years	20	19.6
3−5 years	15	14.7
Between 5 and 10 years	17	16.7
More than 10 years	32	31.4
More than 20 years	18	17.6

**Table 2:** Reasons for drug-taking.

Variable	*n*	%
Most important reason for taking drugs (*n* = 103)		
Peer influence	32	31.1
Feeling bored or depressed	28	27.2
Release pressure	10	9.7
Refreshing	9	8.7
Curious	9	8.7
To avoid feeling discomfort when not taking drugs	4	3.9
Seeking excitement/pleasure	3	2.9
Family influence	2	1.9
Others	6	5.9

**Table 3:** Coding structure of narratives of DATC participants.

Themes	Subthemes
Intrinsic motivation: Competence	Reasons for taking drugs: → Lack of self-worth/confidence/meaning of life Reasons for quitting drugs: → Regain hope and purpose of life when having concrete life goals → Being able to self-discipline
Intrinsic motivation: Autonomy	Reasons for taking drugs: → More willing to take drugs than quitting drugs Reasons for quitting drugs: → More willing to quit drugs than taking drugs → Having understood the negative consequences of taking drugs (e.g., unattractive appearance, hamper the relationship with significant others such as family and friends)
Intrinsic motivation: Relatedness	Reasons for taking drugs: → Negative emotions (e.g., sad, upset) aroused from the poor relationships (e.g., conflict, lack of communication, generation gap, break-up) with significant others (e.g., parents, spouses) → Drugs as psychological substitutes to seek relief, comfort, and satisfaction → Need for affiliation (e.g., negative peer influence, influence by parents) Reasons for quitting drugs: → Receiving care and support (i.e., intimacy) from the significant others → Achieving psychological/spiritual sustenance from religion and life goals
Extrinsic motivation	Reasons for taking drugs: → Strict/severe parental control and discipline/parental pressure (e.g., loss of freedom) Reasons for quitting drugs: → Strict/severe parental control and discipline/parental pressure

## Appendix A. Semi-Structured Interview Guideline

1. When did you first start using drugs? What was the reason for doing so?
2. What was the reason for your need to continue using drugs? What situation would you be triggered to think of using drugs?
3. What does drug use mean to you? What do you gain from using drugs? Do you lose anything from using drugs?
4. If you were to talk about your most painful experience of drug use, what would it be?
5. How much does work/life stress affect your drug use?
6. How much does your friend, boy/girlfriend, and family affect your drug use?
7. Is drug use related to being deprived of faith or spirituality? What do you think?
8. When you have urges to use drugs, what do you feel and what would you do?
9. What is the most helpful method to help you handle the urges? What are the reasons when you cannot handle your urges?
10. Who can help you resist from relapsing?
11. In the past, have you tried to quit drug use? What methods have you tried? What was the longest period you were able to abstain? How could you sustain it?
12. To you, what is the best reason or method to quit drug use?
